# Nonlinear Optimization-Based Device-Free Localization with Outlier Link Rejection

**DOI:** 10.3390/s150408072

**Published:** 2015-04-07

**Authors:** Wendong Xiao, Biao Song, Xiting Yu, Peiyuan Chen

**Affiliations:** School of Automation & Electrical Engineering, University of Science and Technology Beijing, Beijing 100083, China; E-Mails: songbiao7664@163.com (B.S.); 13671309216@163.com (X.Y.); chenpeiyuan1994@126.com (P.C.)

**Keywords:** device-free localization (DFL), outlier link rejection, nonlinear optimization, clustering centriod

## Abstract

Device-free localization (DFL) is an emerging wireless technique for estimating the location of target that does not have any attached electronic device. It has found extensive use in Smart City applications such as healthcare at home and hospitals, location-based services at smart spaces, city emergency response and infrastructure security. In DFL, wireless devices are used as sensors that can sense the target by transmitting and receiving wireless signals collaboratively. Many DFL systems are implemented based on received signal strength (RSS) measurements and the location of the target is estimated by detecting the changes of the RSS measurements of the wireless links. Due to the uncertainty of the wireless channel, certain links may be seriously polluted and result in erroneous detection. In this paper, we propose a novel nonlinear optimization approach with outlier link rejection (NOOLR) for RSS-based DFL. It consists of three key strategies, including: (1) affected link identification by differential RSS detection; (2) outlier link rejection via geometrical positional relationship among links; (3) target location estimation by formulating and solving a nonlinear optimization problem. Experimental results demonstrate that NOOLR is robust to the fluctuation of the wireless signals with superior localization accuracy compared with the existing Radio Tomographic Imaging (RTI) approach.

## 1. Introduction

Wireless localization has found extensive applications in Smart Cities in areas such as healthcare at home and hospitals (e.g., for location detection and behavior analysis of patients and old people), location-based services in smart spaces (airports, shopping centres, touristic sites *etc.*), emergency response (e.g., location detection of firemen in a building on fire), comfortable and energy saving designs for indoor environment monitoring and control, as well as intrusion detection, localization and tracking in infrastructure security.

Various wireless localization techniques have been developed, e.g., based on GPS [1], ultrasound [2], infrared [3], and radio-frequency (RF) [4]. In such techniques, the target must be equipped with an electronic device. Recently device-free localization (DFL), generally based on wireless network RF signal measurements, where the target does not need to have any attached electronic device, has attracted tremendous interest [5]. In DFL, wireless devices are used as sensors that sense the target by transmitting and receiving wireless signals collaboratively, and the target location is estimated by detecting and measuring the RF signal variations induced by the target.

DFL can be implemented in indoor and outdoor environments and remains a challenging problem, mainly due to the uncertain and dynamic wireless propagation environment that suffers from non-line-of-sight, multi-path, and fading phenomena [6,7,8,9,10]. Some links, even without the presence of the targets, may be found to display large received signal strength (RSS) changes and cause erroneous target detection. If such links are used in the location estimation, the accuracy of the localization algorithm will be degraded significantly. However, most existing DFL approaches, such as the Radio Tomographic Imaging (RTI) approach [7,8,9] and the geometrical approach [11,12], do not consider such links explicitly. Although [13] considers the outlier link rejection, only the links without intersections with all the other links are rejected and this may still keep wrong links in many situations.

In this paper, we consider the DFL problem for a single target and will propose a novel optimization approach with outlier link rejection (NOOLR) for DFL by a clustering [14]-based outlier link rejection strategy, followed by formulating and solving a nonlinear optimization problem. In summary, the contributions of this paper are three-fold:

(1) We present a clustering-based technique to reject the outlier links to reduce the effect of large wireless noise on the DFL.
(2) We formulate the DFL problem with a nonlinear optimization model and solve it by the convex optimization approach.
(3) We evaluate NOOLR based on the experimental data provided by the University of Utah, and compare it with the RTI model [7].

The paper is organized as follows: the related work is introduced in Section 2. A motivating application scenario for elder care is described in Section 3. The details of the proposed approach are addressed in Section 4. Experimental results are reported in Section 5 and finally, conclusions and future work are given in Section 6.

## 2. Related Work

A number of DFL approaches have been proposed, including fingerprinting approaches based on differential RSS measurements, geometric approach, RTI approach, compressive sensing approach, and Bayesian approach.

Youssef *et al*., proposed a fingerprinting approach for Wi-Fi-based DFL [15,16]. A radio map is built in the offline phase by recording the differential RSS measurements of links when the target is located at reference points with known locations. In the online phase, the location of the target is estimated by comparing the measured differential RSS signals of the links with the offline radio map. Although the performance of this approach is acceptable, a tedious calibration procedure is required. It is also challenging to efficiently build the radio map.

As a typical geometric approach, Zhang *et al*., proposed a signal dynamics model to obtain the property of the changing RSS behaviors in WSNs, and three tracking algorithms for the midpoint, the intersection, and the best-cover geometric calculations. Although the best-cover algorithm can achieve high accuracy, it also needs a tedious calibration step [11,12].

Wilson and Patwari proposed the RTI approach based on differential RSS link measurements [7,8,9] between the measured RSS measurements of links and the RSS measurements of links without the target via the reconstruction of the tomography image for the locations of the target, and formulated the DFL problem as a linear ill-posed inverse problem, then solved it by the regularization method. Kaltiokallio *et al*., presented an on-line recalibration method that allows the system to adapt to the changes in the radio environment [17].

As compressive sensing can deal with the space-domain sparse information, which is a specific feature of the RTI approach for DFL, Wang *et al*., applied compressive sensing to tackle the ill-posed inverse problem in signal reconstruction in DFL [18,19,20,21].

To reduce the complex computation overhead in solving the ill-posed inverse problem, a Bayesian grid approach (BGA) is proposed in [22] by utilizing the observation information of the shadowed links, the prior information of the previous estimations, and the constraint information of the non-shadowed links. Savazzi *et al*., proposed a joint model based on the theory of diffraction to deal with the average path-loss and the fluctuations of the RSS induced by the moving target, and derive a novel stochastic Bayesian model for the real-time estimation of the target location [23]. Overall the above-mentioned approaches do not consider the negative effects of the outlier links for the localization, and the localization accuracy is limited.

## 3. Motivating Application Scenario in Smart Aging

**Figure 1:**
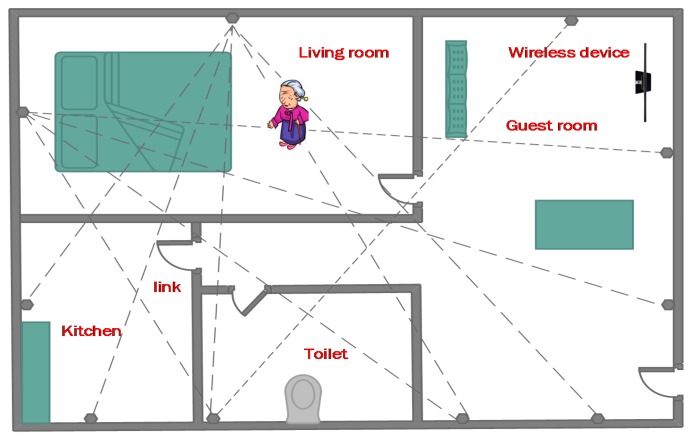
DFL application scenario for aging at home.

With the constantly increasing aging population with cognitive deficiencies, insuring the autonomy of the elderly at home has become a priority for assisting people and improving their quality of life. The ability to monitor the elder’s activities and provide timely assistance depends highly on the location information for the elder inside the home. Referring to Figure 1 as a motivating scenario, based on the location information, the elder person’s activities can be monitored. For example, he/she can be advised to do more movement exercises if needed. If abnormalities are detected (e.g., suddenly falling down, staying in bed or in toilet for an unusually long time), alarms can be triggered to inform caregivers and family members, while providing the location information.

The elder can be localized by traditional technologies such as cameras, RFID, WiFi, or some other wearable devices. However these may introduce heavy burdens for the elders to carry such devices, and cause privacy and comfort problems. Therefore it is desirable for the elder people not to be equipped with any electronic device, and implement DFL for the localization demands.

## 4. The Details of the Proposed Approach

In this section, we give the detailed description of NOOLR for DFL. It consists of three key strategies implemented sequentially, including the affected link identification by differential RSS detection, outlier link rejection via positional relationships of links, as well as the target location estimation by formulating and solving a nonlinear optimization problem.

### 4.1. Affected Link Identification

Suppose there are *N* wireless devices used as transmitters or receivers for detecting the target. If wireless device i and wireless device *j* can communicate with each other directly, then we denote the link by $L_{n}\left(i,j\right)$ and $L_{n}\;$ for simplicity, with *n* being the ID of the link. We assume the total number of links in the system is P.

Let $X_{i}\left(x_{i},y_{i}\right)$ denote the location of the wireless device i in the Cartesian coordinates, $RSS_{n}^{0}\;$ and $RSS^{0}=$ $\left\{RSS_{1}^{0},\;RSS_{2}^{0},\ldots ,\;RSS_{P}^{0}\right\}$ denote the RSS measurements of link n and the RSS measurements of all links respectively when the monitoring area is vacant. Also let $RSS_{n}$ and $RSS=\left\{RSS_{1},\;RSS_{2},\ldots ,\;RSS_{P}\right\}$ denote the RSS measurements of link n and the RSS measurements of all links respectively when the target enters the monitoring area.

The difference between the RSS of the link *n* with the target and without the target in the monitoring area is:

(1) $$∆RSS_{n}=\;RSS_{n}-\;RSS_{n}^{0}$$

The main contribution of the $∆RSS_{n}$ of link *n* is due to the presence of the target and the noise. We call the links with large differential RSS induced by the target as the affected links.

Finding the affected links is essential for the localization algorithms. In the offline phase, the radio map can be built by collecting the RSS measurements of links, *i.e.*, $RSS^{0}=\left\{RSS_{1}^{0},\;RSS_{2}^{0},\ldots ,\;{\text{RSS}}_{\text{P}}^{0}\right\}$. RSS can be obtained and the differential RSSs of links ($∆RSS=\left\{∆RSS_{1},\;∆RSS_{2},\;\ldots ,\;∆RSS_{P}\right\}\;$) can be calculated. A negative scalar threshold γ is used to identify whether a link is an affected link and whether the target is present in the monitoring area [24]. Normally if the link *n* is affected by the target, $∆RSS_{n}$ will be less than the threshold γ [25]. The state of the link *n* can be calculated by:

(2) $${\text{s}}_{n}=\{\begin{array}{c} 1,\;if\;∆RSS_{n}\leq \gamma \;\\ 0,\;if\;∆RSS_{n}\geq \gamma \;\; \end{array}$$

If ${\text{s}}_{n}=1$, then link *n* will be considered as the affected link. The states of all links $s=\left\{{\text{s}}_{1},{\text{ s}}_{2},\;\ldots ,{\text{s}}_{P}\right\}$ can be obtained according to Equation (2).

### 4.2. Outlier Link Rejection

**Figure 2:**
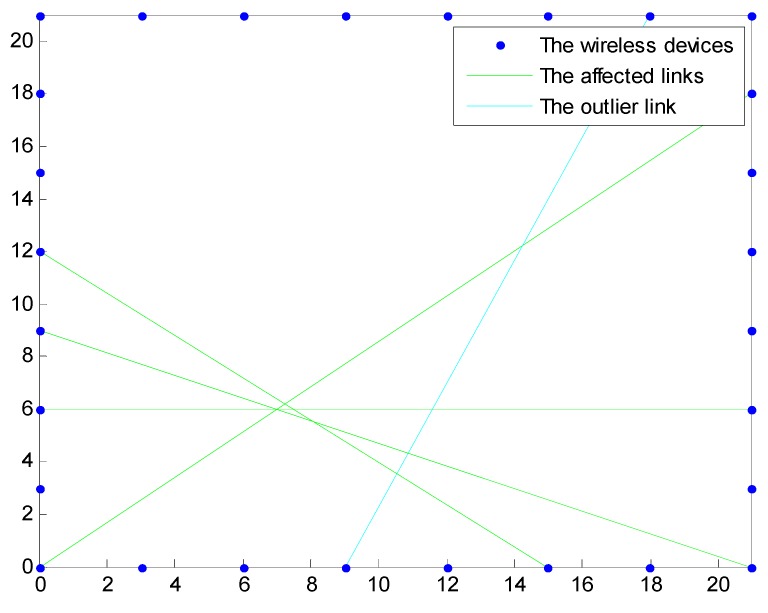
Affected links and outlier links.

Due to the uncertain wireless propagation environment, some links associated with large noise but that are far away from the target may be incorrectly identified as the affected links, and significantly degrade the accuracy of the localization algorithms. They are considered as the outlier links in this paper and shall be rejected in the localization approach. As shown in Figure 2, the affected link that can detect the target shall be around the target and near to each other, and an outlier link is separated and far away from those affected links around the target.

In NOOLR, the outlier links are identified and rejected by clustering the identified affected links by transforming the positional relationship of links into one-dimensional distance information. The affected links can be obtained from the states of links $s=\left\{s_{1},\;s_{2},\;\ldots ,s_{P}\right\}$. We assume the number of affected links is *m*. The number of intersections of the affected links is less than or equal to m(m−1)/2. We assume the number of intersections is R. Because the coordinates of wireless devices are known, the coordinates of intersections can be calculated. Here we use $X_{i,j}\left(x_{i,j},y_{i,j}\right)$ to denote the coordinates of the intersection of link i and link j.

*Definition 1*. The center of the intersections ($x_{c},\;y_{c}$) is the average coordinates of the intersections of the affected links, *i.e.*:

(3) $$\{\begin{array}{c} x_{c}=\;\frac{\sum_{i=1}^{P}\sum_{j=1}^{P}{\text{s}}_{i}{\text{s}}_{j}x_{i,j}}{\text{R}}\\ y_{c}=\;\frac{\sum_{i=1}^{P}\sum_{j=1}^{P}{\text{s}}_{i}{\text{s}}_{j}y_{i,j}}{\text{R}} \end{array}$$

The geometrical positional relationship of links is transformed to the distance values $dis=\left\{dis_{1},dis_{2},\ldots ,dis_{\text{m}}\right\}$ where $dis_{n}$ denotes the distance between the affected link *n* and the center of the intersections ($x_{c},\;y_{c}$). The distance between the outlier link and the center of the intersections is always greater than the distance between the affected links and the center of the intersections. The variance of the distance values is used to detect the outlier links, and the variance of the distance values $V_{dis}$ is:

(4) $$V_{dis}=\frac{\sum_{i=1}^{m}{\left(dis_{i}-dis_{-}\right)}^{2}}{m-1}$$

where $dis_{-}$ is the average value of dis. A threshold of variance (δ) is selected empirically to detect the outlier links. Then the outlier state of links can be calculated as:

(5) $$o=\{\begin{array}{l} 1,\;\;V_{dis}\geq \delta \\ 0,\;\;otherwise \end{array}$$

**Figure 3:**
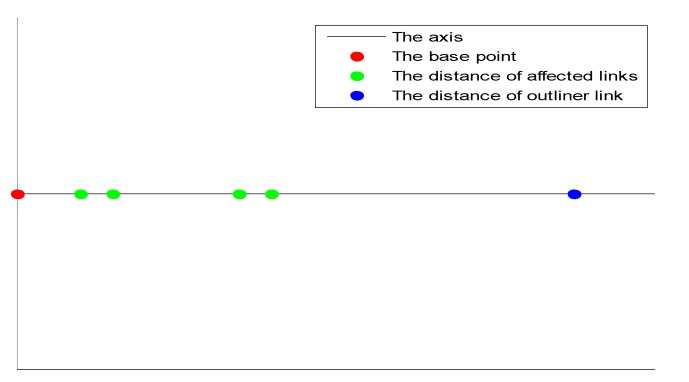
Distribution of the mapped distances.

If o=0, we consider that there exist no outlier links in the affected links. If o=1, the outlier links are detected and shall be rejected from the affected links. The center of the intersections and dis are mapped to 1-dimension space with the center of the intersections as the origin, as illustrated in Figure 3. Clustering will be performed to identify and reject the outlier links as described below.

Firstly, the distances associated with the links $dis=\left\{dis_{1},dis_{2},\ldots ,dis_{\text{m}}\right\}$ are considered as a data set $\left\{x_{1},x_{2},\;\ldots ,x_{m}\right\}$ and clustered by the K-means algorithm [26]. Two clusters are generated for the affected links and the outlier links, respectively, and their corresponding centroids are calculated. Then, each data in the data set is clustered to the nearest centroid, and the centroids will be updated by recalculating the average data in each cluster [26,27]. With the new centroids, a new loop is formed and each data is associated to the nearest new centroid. Finally, the loop stops until the centroids are not changed any more. This approach aims at minimizing an objective function described as:

(6) $$\text{J}=\;\overset{2}{\underset{j=1}{\sum }}\overset{\text{m}}{\underset{i=1}{\sum }}∥x_{i}^{j}-c_{j}{∥}^{2}$$

where $x_{i}^{j}$ is the associated data in the data set, and $c_{j}$ is the centroid. Because the distance between the center of the intersections and an outlier link is larger than the distance between the center of the intersections and the affected link, the centroid with the larger coordinate should belong to the outlier links. Finally, the cluster with the larger centroid is associated with the set of the outlier links. The process that detect and reject outlier links is summarized in Algorithm 1.

**Algorithm 1** Detection and rejection of outlier linksGet the $∆RSS^{t}$ according to Equation (1)Get the state of links according to Equation (2)If there are the effected linksCalculate the coordinates of the intersectionsCalculate the center of the intersections (${\text{x}}_{\text{c}},{\text{ y}}_{\text{c}}$) according to Equation (3)Get the variance of the distances according to Equation (4)Estimate the outlier state of links according to Equation (5)If there are the outlier links (outlier state is 1)Cluster the affected links use K-means algorithm based on the distance values $$dis=\left\{dis_{1},dis_{2},\ldots ,dis_{\text{m}}\right\}$$The links in the cluster with the larger centroid coordinate is the outlier links,Eliminate the outlier links.EndEnd

### 4.3. Nonlinear Optimization for Location Estimation

*Definition 2*: The projection $Q_{in}$ from the position i to link n is the point on link $L_{n}$ which is closest to position i, and the distance from position i to link *n* is the distance $D_{in}\;$between position i and the projection $Q_{in}$. If $X_{i}\;$is on link $L_{n}$, the projection is itself and $D_{in}=\;0$, otherwise the distance $D_{in}$ can be calculated by:

(7) $$D_{in}\left(x_{0},\;y_{0}\right)=\min_{{\text{X}}_{\text{j}}\epsilon L_{n}}\text{dis}\left({\text{X}}_{\text{i}},{\text{ X}}_{\text{j}}\right)$$

where $X_{i}$ is the coordinate of the position i. For $X_{i}$ and $X_{j}$ with coordinates $\left(x_{i},y_{i}\right)$ and $\left(x_{j},y_{j}\right)$, their distance $dis\left(X_{i},\;X_{j}\right)$ is given by:

$$dis\left(X_{i},\;X_{j}\right)=\sqrt{{\left(x_{i}-x_{j}\right)}^{2}+{\left(y_{i}-y_{j}\right)}^{2}}$$

By the clustering algorithm mentioned earlier, the affected links with outlier link rejection can be obtained. In this section, we consider the affected links as the affected links without outlier links, and the number of affected links is *M*.

**Figure 4:**
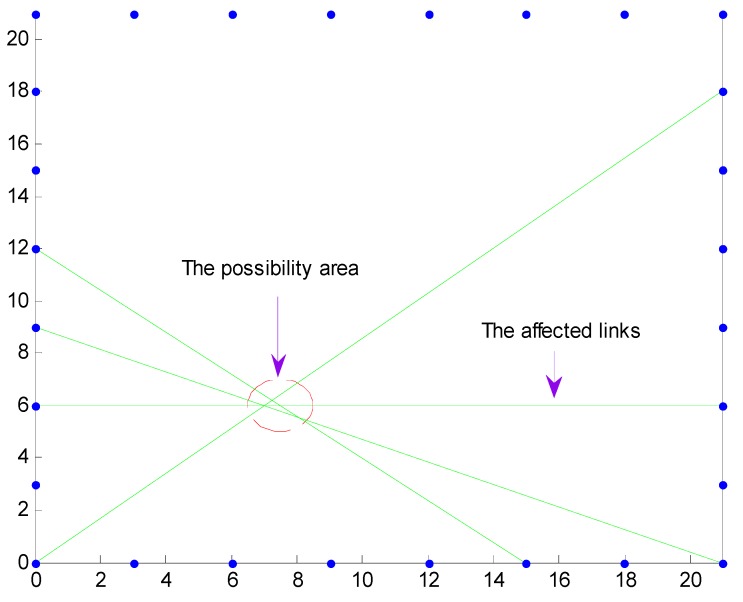
Possible location area of the target.

The main contribution for the RSS difference of an affected link is caused by the target. Thus, the target must be nearby the affected links or on the affected links. As shown in Figure 4, the affected links intersect with each other, and the target location shall be inside the red circle. We assume the target location is $X_{0}\left(x_{0},\;y_{0}\right)$. The projection $Q_{0n}$ from target location $X_{0}\left(x_{0},y_{0}\right)$ to the affected link $L_{n}$ ($L_{n}\in s_{als}$) and the distance $D_{0n}$ can be obtained. The DFL problem can be translated into an optimization problem according to the relationship between the target location and the affected links. The point with the minimum sum of distance to the affected links is used as the estimation of the target location. Mathematically, the optimal target location $X_{0}^{*}\left(x_{0,\;}^{*}\;y_{0,\;}^{*}\right)$ is obtained by:

(8) $$X_{0}^{*}\left(x_{0,\;}^{*}y_{0,\;}^{*}\right)=argmin_{\left(x_{0},\;y_{0}\right)}\overset{M}{\underset{i=1}{\sum }}D_{0i}\left(x_{0},\;y_{0}\right)$$

For a given affected link *i* of the transmitter and receiver with known coordinates, assume its associated linear equation is expressed by:

(9) $$k_{i}x+c_{i}y+d_{i}=0$$

Then the distance $D_{0i}$ can be calculated according to Equations (7) and (9) by:

(10) $$D_{0i}\left(x_{0},\;y_{0}\right)=\;\frac{\left|k_{i}x_{0}+c_{i}y_{0}+d_{i}\right|}{\sqrt[{2}]{{k_{i}}^{2}+{c_{i}}^{2}}}$$

Then we can re-write Equation (8) as:

(11) $$X_{0}^{*}\left(x_{0,\;}^{*}y_{0,\;}^{*}\right)=argmin_{\left(x_{0},\;y_{0}\right)}\overset{M}{\underset{i=1}{\sum }}\frac{\left|k_{i}x_{0}+c_{i}y_{0}+d_{i}\right|}{\sqrt[{2}]{{k_{i}}^{2}+{c_{i}}^{2}}}$$

As the above nonlinear optimization problem is difficult to solve. We replace $D_{0i}\;$with ${\left(D_{0i}\right)}^{2}$ and re-formulate DFL problem as the following nonlinear optimization problem:

(12) $$X_{0}^{*}\left(x_{0,\;}^{*}y_{0,\;}^{*}\right)=argmin_{\left(x_{0},\;y_{0}\right)}\overset{M}{\underset{i=1}{\sum }}\frac{{\left(k_{i}x_{0}+c_{i}y_{0}+d_{i}\right)}^{2}}{{k_{i}}^{2}+{c_{i}}^{2}}$$

Now we define some notations in Table 1 and give two important theorems.

**Table 1:** The notations.

Notations	Description
dom f	The domain of function f
∇f	The first-order partial derivatives of x
$\nabla f_{x}$	The Hessian matrix of function f
$\text{det}\nabla^{2}f$	The value of Hessian matrix $\nabla^{2}f$

*Theorem 1* [28]: If the function f is twice differentiable, that is, its Hessian or second derivative $\nabla^{2}f$ exists at each point in dom f, which is open, then f is convex if and only if dom f is convex and its Hessian is positive semidefinite: for all x∈dom f:

$$\nabla^{2}f\geq 0$$

*Theorem 2* [28]: For the unconstrained optimization:

minimize f(x)

where f is convex and differentiable, there exists an optimal point $x^{*}$, and the necessary and sufficient condition for a point $x^{*}$ to be optimal is:

$$\nabla f\left(\;x^{*}\right)=0$$

Denote the following nonlinear function:

(13) $$f\left(x_{0},\;y_{0}\right)=\overset{M}{\underset{i=1}{\sum }}\frac{{\left(k_{i}x_{0}+c_{i}y_{0}+d_{i}\right)}^{2}}{{k_{i}}^{2}+{c_{i}}^{2}}$$

In the DFL problem, the dom f is the monitoring area and we assume it is convex in this paper. The Hessian matrix of *f* is:

(14) $$\nabla^{2}f=\;\left(\begin{array}{cc} \overset{M}{\underset{i=1}{\sum }}\frac{2{k_{i}}^{2}}{{k_{i}}^{2}+{c_{i}}^{2}} & \overset{M}{\underset{i=1}{\sum }}\frac{2k_{i}c_{i}}{{k_{i}}^{2}+{c_{i}}^{2}}\\ \overset{M}{\underset{i=1}{\sum }}\frac{2k_{i}c_{i}}{{k_{i}}^{2}+{c_{i}}^{2}} & \overset{M}{\underset{i=1}{\sum }}\frac{2{c_{i}}^{2}}{{k_{i}}^{2}+{c_{i}}^{2}} \end{array}\right)$$

It is obvious that$\;\overset{M}{\underset{i=1}{\sum }}\frac{2{k_{i}}^{2}}{{k_{i}}^{2}+{c_{i}}^{2}}\geq 0$, and $det\nabla^{2}f/4=\;\overset{M}{\underset{i=1}{\sum }}\frac{{k_{i}}^{2}}{{k_{i}}^{2}+{c_{i}}^{2}}\times \;\overset{M}{\underset{i=1}{\sum }}\frac{{c_{i}}^{2}}{{k_{i}}^{2}+{c_{i}}^{2}}-\;{\left(\overset{M}{\underset{i=1}{\sum }}\frac{k_{i}c_{i}}{{k_{i}}^{2}+{c_{i}}^{2}}\right)}^{2}$. We use the notations$\;A_{i}^{2}$ and $B_{i}^{2}\;$ to stand for $\frac{{k_{i}}^{2}}{{k_{i}}^{2}+{c_{i}}^{2}}$ and $\frac{{c_{i}}^{2}}{{k_{i}}^{2}+{c_{i}}^{2}}$ respectively, then $\frac{k_{i}c_{i}}{{k_{i}}^{2}+{c_{i}}^{2}}$ can be represented as $A_{i}B_{i}$ and we have:

(15) $$det\nabla^{2}f/4=\;\overset{M}{\underset{i=1}{\sum }}A_{i}^{2}\times \overset{M}{\underset{i=1}{\sum }}B_{i}^{2}-{\left(\overset{M}{\underset{i=1}{\sum }}A_{i}B_{i}\right)}^{2}$$

The equation by factorization can be written as:

(16) $$det\nabla^{2}f/4=\overset{M}{\underset{i=1}{\sum }}\overset{M}{\underset{j=1}{\sum }}{\left(A_{i}B_{i}-A_{j}B_{j}\right)}^{2}$$

and $det\nabla^{2}f/4\geq 0$*.*

Because $\overset{M}{\underset{i=1}{\sum }}\frac{2{k_{i}}^{2}}{{k_{i}}^{2}+{c_{i}}^{2}}\geq 0$ and $det\nabla^{2}f\geq 0$*,* the Hessian matrix $\nabla^{2}f$ is positive semidefinite, and dom f is convex, we know that *f* is convex according to Theorem 1.

As f is convex and differentiable, according to Theorem 2, the optimal point $X_{0}^{*}\left(x_{0,\;}^{*}y_{0,\;}^{*}\right)$ for problem Equation (12) exists and can be calculated by:

(17) $$\{\begin{array}{c} \nabla f_{x}=0\\ \nabla f_{y}=0 \end{array}$$

where $\nabla f_{x}=\;\overset{M}{\underset{i=1}{\sum }}\frac{2k_{i}\left(k_{i}x_{0}+c_{i}y_{0}+d_{i}\right)}{{k_{i}}^{2}+{c_{i}}^{2}},\;\nabla f_{y}=\;\overset{M}{\underset{i=1}{\sum }}\frac{2c_{i}\left(k_{i}x_{0}+c_{i}y_{0}+d_{i}\right)}{{k_{i}}^{2}+{c_{i}}^{2}}.$

The optimal point $X_{0}^{*}\left(x_{0,\;}^{*}y_{0,\;}^{*}\right)\;$of Equations (17) is the estimation of the target location. Now we give the complexity analysis for NOOLR. Assume that the number of loops in the K-means algorithm is q, and the number of intersections of the initial affected links is R as mentioned earlier, the computational complexity of Algorithm 1 is O(qR), thus according to Equation (17), the computational complexity of the nonlinear optimization based NOOLR approach is O(qR).

## 5. Experimental Evaluation

### 5.1. Experimental Setup

**Figure 5:**
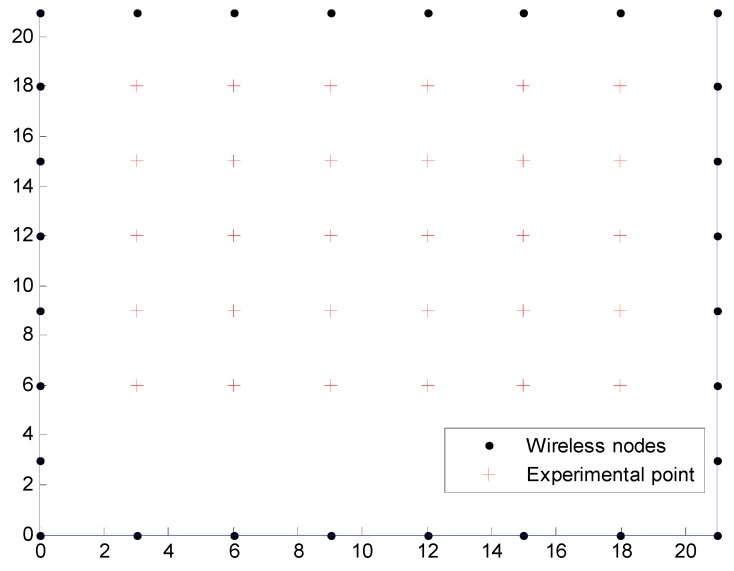
The experimental setup.

To evaluate the performance of the nonlinear optimization model for the DFL, we performed some experiments based on the experimental data that can be acquired [29] from the SPAN Lab of the University of Utah (Salt Lake City, UT, USA). There are 28 wireless devices and a Crossbow base station device, and the total number of links is 378. Each device operates in the 2.4 GHZ frequency band, and uses the IEEE 802.15.4 standard for communication. The base station node listens to all network traffic, then feeds the data to a laptop computer via a USB port for the processing of NOOLR. The RSS of each link is an averaged value of the RSS measurements from bi-directional transmissions. Each wireless device is placed three feet apart along the perimeter of a 21 × 21 feet square, surrounding a total area of 441 square feet, and is placed on a stand at three feet off the ground. In the experiment, the target is a person with the height 1.85 m and the weight 88 kg. As shown in Figure 5, 30 testing locations are selected to compare the nonlinear optimization model for the DFL with RTI reconstruction [7].

In this experiment, the system is calibrated by collecting RSS measurements while the network is vacant from targets. The time for calibration is 30 s. The time for data initialization which processes the calibration data is 2.0759 seconds. We perform experiments to test the running time for NOOLR. The running time $t_{total\_time}$ contains two portions, including the time $t_{data\_time}$ for processing the real time data and the time $t_{NOOLR\_time}$ for running NOOLR.

We repeat the trials for 30 times, and the statistic results are summarized in Table 2.

**Table 2:** Running time for NOOLR in the experiment.

Description	Mean(s)	Worst(s)	Best(s)
$t_{data\_time}$	1.5420	1.6051	1.4917
$t_{NOOLR\_time}$	0.0204	0.1244	0.0052
$t_{total\_time}$	1.5624	1.6973	1.5130

NOOLR can achieve averaged, worst and best times of 0.0204 s, 0.1244 s and 0.0052 s, which is very low, the average, the worst and the best times for the total running time is 1.5624 s, 1.6973 s and 1.5130 s, respectively, which is acceptable for target location estimation.

In RTI, the monitored area is divided into voxels, aggregately all voxels contribute to the RSS changes of each transmitter-receiver link in the network. The weights of all voxels are computed according to their impacts on all the links, and the voxel with the minimum weight is considered as the location estimation of the target. The RTI reconstruction for obtaining the weights of the voxels uses H1 regularization with the parameters listed in Table 3.

**Table 3:** The parameters of RTI.

Parameter	Value	Description
∆p	0.5	Pixel width(feet)
E	0.01	Width of weighting ellipse (feet)
A	5	Regularization parameter

The NOOLR parameters are listed in Table 4. As the analytical relationship between the parameters and the system performance of NOOLR is not clear, we use the trial and error method to determine the parameters.

**Table 4:** The parameters of NOOLR.

Parameter	Value	Description
γ	−6	The threshold for affected link detection
δ	0.5	The threshold for the variance

**Figure 6:**
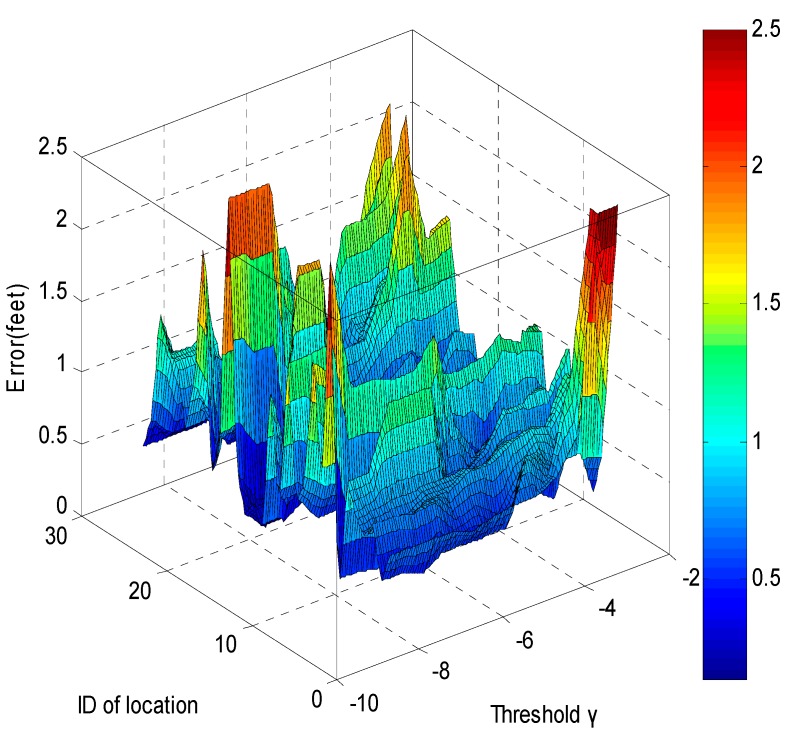
The errors when γ changes.

**Figure 7:**
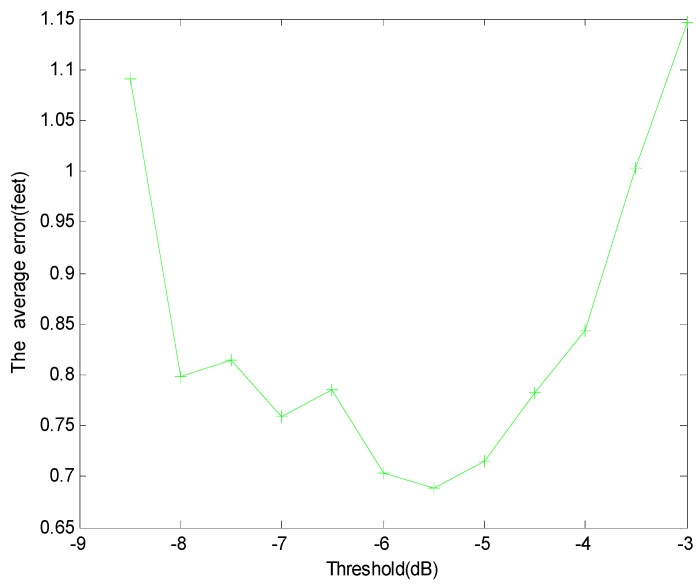
Average errors for different γ

The Euler distance between the “true” location of the target and the estimated location of the target is used as the localization error. The localization errors against different thresholds γ are shown in Figure 6 and Figure 7 when the threshold for the variance δ is 0.5. If γ<−8 dB, there may be not enough affected links for localization, and the average error of the algorithm increases sharply.

**Figure 8:**
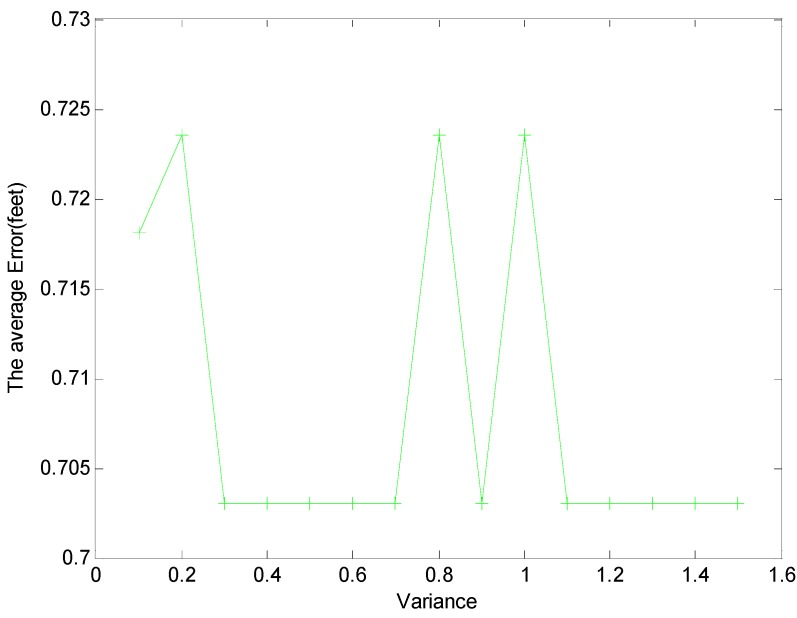
Average errors for the different thresholds of variance (δ).

Meanwhile, from Figure 7, due to the uncertain wireless propagation environment, if γ≥−5.5 dB, some links with large noise are incorrectly identified as the affected links, the average error of localization will also increase. The localization errors against different thresholds of variance δ are shown in Figure 8 when the threshold γ is −6. If δ<0.3, the outlier state of links $o_{t}$ may be incorrectly detected, and the error of localization increases. Nevertheless, if δ≥0.7, an outlier link may be missed, and the error of localization is increased.

### 5.2. Performance Comparison

**Figure 9:**
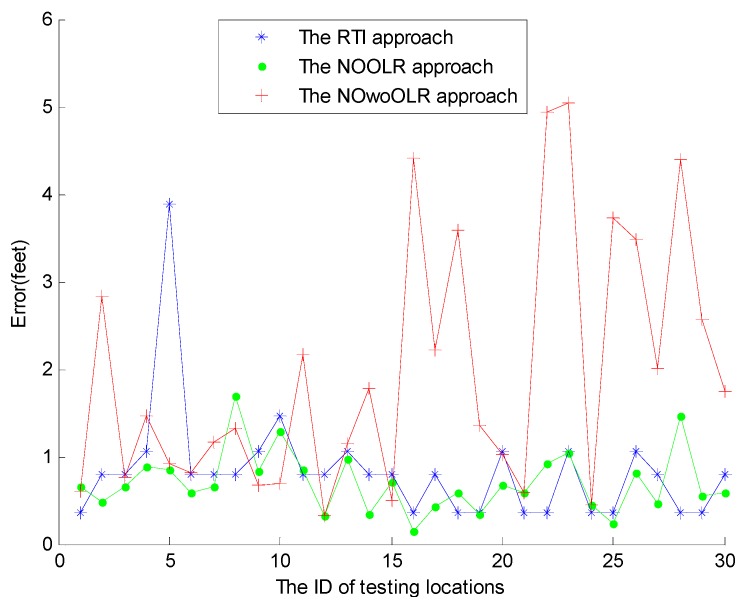
Performance comparison of NOOLR, NOwoOLR, and RTI.

To evaluate the performance of NOOLR, we compare it with the scheme of nonlinear optimization without outlier link rejection (NOwoOLR), and RTI. The detailed comparison results for 30 testing locations are shown in Figure 9 and Table 5.

**Table 5:** Performance comparison of NOOLR , NOwoOLR, and RTI.

Algorithm	Mean (Feet)	Variance	Worst (Feet)	Best (Feet)	Median (Feet)
NOOLR	0.7030	0.1210	1.6861	0.1500	0.6554
NOwoOLR	1.9571	2.1034	5.0493	0.3170	1.4104
RTI	0.8244	0.4262	3.8891	0.3536	0.7905

The mean localization error and the worst localization error of NOOLR are reduced by 10.6%, 50.2%, respectively, from RTI, which shows a significant improvement. The best localization error of NOOLR can achieve is 0.1197 feet. Statistically the performance of NOOLR is the best, followed by RTI and NOwoOLR, this is because RTI uses all the links, NOwoOLR uses the affected links, and NOOLR uses the affected links with outlier link rejection to calculate the target locations, and the different impacts of the noises and outlier links are reflected in the localization accuracy. The variance of the localization errors reveals the robustness of the DFL systems. The smaller variance of NOOLR illustrates that NOOLR is more robust and reliable. It is obvious that the proposed nonlinear optimization model has better performance than RTI.

Meanwhile it is obvious that NOwoOLR has the worst performance. Outlier links are not eliminated in NOwoOLR. RTI utilizes the RSS measurements of all links at time t. The outlier links can degrade the accuracy of RTI. After the outlier links are rejected, it is beneficial for the localization algorithms using the nonlinear optimization model.

## 6. Conclusions

This paper proposes a novel nonlinear optimization based approach for DFL called NOOLR as a valuable localization approach for Smart City applications. To decrease the negative impacts of the large noise of the wireless links, outlier link detection and rejection are suggested by considering the geometrical relationship of links and utilizing a the clustering algorithm. A nonlinear optimization model is formulated for the location estimation to minimize the distance of the target location to the affected links. Experimental results show the superior localization performance of NOOLR against RTI. As future work, DFL for multiple targets and a case Smart City study will be considered as important problems to be solved.
